# Automatic quantitative measurement of left atrial pressure using mitral regurgitation spectrum: clinical study on comparison with floating catheter

**DOI:** 10.1186/s40001-022-00849-y

**Published:** 2022-10-28

**Authors:** Yan Jin, Chao-yang Wen, Fengjie Yue, Huishan Wang, Liancheng Yin, Yang Zhao, Keming Mao, Fangran Xin

**Affiliations:** 1 Department of Cardiovascular Surgery, General Hospital of Northern Theater Command, No. 83, Wenhua Road, Shenhe District, Shenyang, 110016 Liaoning China; 2 Department of Ultrasound, Peking University International Hospital, Beijing, China; 3 Department of Ultrasound Medicine, Xiangyang Central Hospital, Affiliated Hospital of Hubei University of Arts and Science, Xiangyang, Hubei China; 4 College of Software, Northeastern University, Shenyang, Liaoning China

**Keywords:** Left atrial pressure, Pulmonary arteriole wedge pressure, Mitral regurgitation spectrum, Deep learning model of big data training

## Abstract

**Introduction:**

To explore how to measure LAP_Eq_ accurately and quantitatively, that is, the left atrial pressure (LAP) measured and calculated by equation method using mitral regurgitation spectrum.

**Methods:**

The mitral regurgitation spectrum, pulmonary arteriolar wedge pressure (PAWP) and invasive arterial systolic pressure of radial artery of 28 patients were collected simultaneously, including 3 patients with rheumatic heart disease, 15 patients with mitral valve prolapse and 10 patients with coronary artery bypass grafting, patients with moderate or above aortic stenosis were excluded. LAP_Bp_ (Doppler sphygmomanometer method), LAP_Eq_ (Equation method) and LAP_C_ (Catheter method) were measured synchronously, and the measurement results of the three methods were compared and analyzed. A special intelligent Doppler spectrum analysis software was self-designed to accurately measure LAP_Eq_. This study had been approved by the ethics committee of the Northern Theater General Hospital (K-2019-17), and applied for clinical trial (No. Chictr 190023812).

**Results:**

It was found that there was no significant statistical difference between the measurement results of LAP_C_ and LAP_Eq_ (*t* = 0.954, *P* = 0.348), and significant correlation between the two methods [*r* = 0.908(0.844, 0.964), *P* < 0.001]. Although the measurement results of LAP_C_ and LAP_BP_ are consistent in the condition of non-severe eccentric mitral regurgitation, there are significant differences in the overall case and weak correlation between the two methods [*r* = 0.210, (−0.101, 0.510), *P* = 0.090]. In MVP patients with P1 or P3 prolapse, the peak pressure difference of MR was underestimated due to the serious eccentricity of MR, which affected the accuracy of LAP_BP_ measurement.

**Conclusions:**

It was shown that there is a good correlation between LAP_Eq_ and LAP_C_, which verifies that the non-invasive and direct quantitative measurement of left atrial pressure based on mitral regurgitation spectrum is feasible and has a good application prospect.

## Introduction

Left atrial pressure (LAP) is a reliable data reflecting the left ventricular preload, which can correctly reflect the change of blood volume and sensitively reflect the left ventricular end diastolic pressure. It is an important hemodynamic parameter for adjusting the treatment plan of critically ill patients [1]. However, it is almost impossible to directly measure left atrial pressure in clinical practices. At present, pulmonary arteriole wedge pressure (PAWP) measured by floating catheter is used to replace LAP_C_ in clinic. However, the measurement of PAWP is also invasive, and its clinical application is greatly limited [2, 3].

Different degrees of mitral regurgitation (MR) often occurred in normal subjects and patients [4]. At present, there are two methods to quantitatively measure LAP according to mitral regurgitation spectrum. The first one is the “Doppler + sphygmomanometer” method [5, 6], that is, the LAP_BP_ is the left ventricular systolic pressure (*P*) minus the mitral regurgitation pressure difference, ${\text{LAP}}_{{{\text{BP}}}} = P - \Delta P$ If there is no aortic valve and/or left ventricular outflow tract stenosis, the arterial systolic pressure can replace the left ventricular systolic pressure. The second one is proposed by our team. According to Weiss exponential equation ($P = {\text{e}}^{{{{ - t} \mathord{\left/ {\vphantom {{ - t} {\tau + {\text{B}}}}} \right. \kern-\nulldelimiterspace} {\tau + {\text{B}}}}}}$), simplified Bernoulli equation ($\Delta P = 4V^{2}$) and $P = \Delta P + {\text{LAP}}$, Bai [7–10] used mathematical methods to derive “binary linear equations”, which have 2 variables, namely the left ventricular relaxation time constant (*τ*) and LAP, and LAP_Eq_ is measured and calculated by using the decline curve of mitral regurgitation spectrum. The calculation of τ has been verified by animal experiments [11]. In animal experiments, our team has confirmed that there is a good correlation between LAP_Eq_ and LAP_C_ (Catheter measurement) [12].

If a non-invasive, convenient and accurate LAP quantitative measurement method can be developed, the diagnosis and treatment of heart related diseases will enter an accurate stage, which is of significant clinical value. We evaluated the accuracy of two methods of quantitative measurement of left atrial pressure using mitral regurgitation spectrum, and discussed how to measure LAP noninvasively, accurately and quantitatively.

## Methods

### Study design and patients

Patients who needed cardiac surgery accompanied by mitral regurgitation were prospectively selected as the research objects, from June 2020 to Oct 2020. The mitral regurgitation spectrum, pulmonary arteriolar wedge pressure (PAWP) and invasive arterial systolic pressure of radial artery of 28 patients were collected simultaneously, 13 males and 15 females, including 3 patients with rheumatic heart disease, 15 patients with mitral valve prolapse and 10 patients with coronary artery bypass grafting, patients with moderate or severe aortic stenosis were excluded, aged 47–78 years (62.55 ± 7.28 years) and PAWP 7–29 mmHg (15.3 ± 4.9 mmHg). This study had been approved by the ethics committee of the Northern Theater General Hospital (K-2019-17), and applied for clinical trial (No. Chictr 190023812).

### Floating catheter

With the assistance of intravenous anesthesia, endotracheal intubation and ventilator, the floating catheter was placed through the jugular vein and the arterial systolic pressure was measured by radial artery puncture. The position of the floating catheter was determined by transthoracic ultrasound. PAWP measured by floating catheter was used to replace LAP_C_. The A wave after the *P* wave of ECG is generated by the active contraction of left atrium, and the C wave is generated by the closure of mitral valve. The V wave after ECG T wave is generated by left ventricular relaxation and left atrial passive filling during mitral valve opening (the pressure generated by this wave cannot be used as left atrial pressure). Therefore, the pressure measured on the PAWP pressure curve at the end of ECG *P* wave was taken as LAP_C_. The equipment used include: Fl-005 GE anesthesia monitor (GE Healthcare Finland), Edwards 131 F7 floating catheter (Irvine, USA), PTC-6F pressure monitoring catheter (Jingzhou Yihai Technology Co., Ltd.), etc.

### Echocardiography

The patient was in supine position because of perioperative period. All ultrasound examinations were performed by the same echocardiographic doctors with a Philips ultrasound system (Philips iE33 ultrasound machine; Philips Healthcare, Andover Mass). The mitral regurgitation spectrum was collected under CW, and the angle between the sampling line and the mitral regurgitation beam should be less than 15°. Different recording speeds of 100 mm/s or 150 mm/s were selected according to the speed of heart rate to obtain a dull, smooth and complete spectrum. Each Doppler spectrum image was measured three times every other day by two ultrasound doctors in a single blind state, and the average value was taken.

### Formulas of LAP_Eq_

**Fig. 1:**
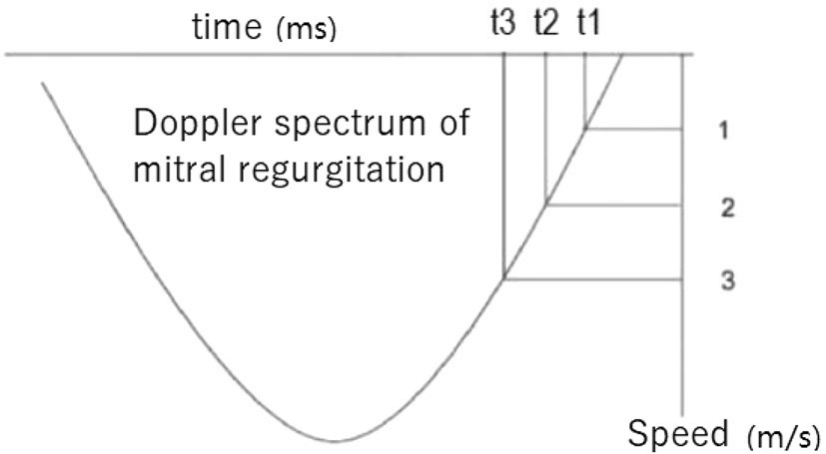
Pressure versus time curve of mitral regurgitation

According to Weiss exponential equation and simplified Bernoulli equation, the left ventricular relaxation time constant (*τ*) can be obtained, $\tau = {P \mathord{\left/ {\vphantom {P {\left( {{{ - {\text{d}}P} \mathord{\left/ {\vphantom {{ - {\text{d}}P} {{\text{d}}t}}} \right. \kern-\nulldelimiterspace} {{\text{d}}t}}} \right)}}} \right. \kern-\nulldelimiterspace} {\left( {{{ - {\text{d}}P} \mathord{\left/ {\vphantom {{ - {\text{d}}P} {{\text{d}}t}}} \right. \kern-\nulldelimiterspace} {{\text{d}}t}}} \right)}}$, where P is the pressure in the left ventricle, and *t* is the time from −d*P*/d*t*_max_, as shown in Fig. 1. The intervals between different speeds were brought in to obtain the following formulas, $\tau = (t1 - t3)/\ln [({\text{LAP}} + 36)/({\text{LAP}} + 4)]$ and $\tau = (t1 - t3)/\ln [({\text{LAP}} + 36)/({\text{LAP}} + 4)]$. Theoretically, LAP can be calculated by measuring the intervals between any two speeds. In order to facilitate calculation and measurement, we selected the time *t*1, *t*2 and *t*3 when the descending branch velocity of mitral regurgitation spectrum was 1 m/s, 2 m/s and 3 m/s, respectively.

### Measurement methods of LAP_Eq_

**Fig. 2:**
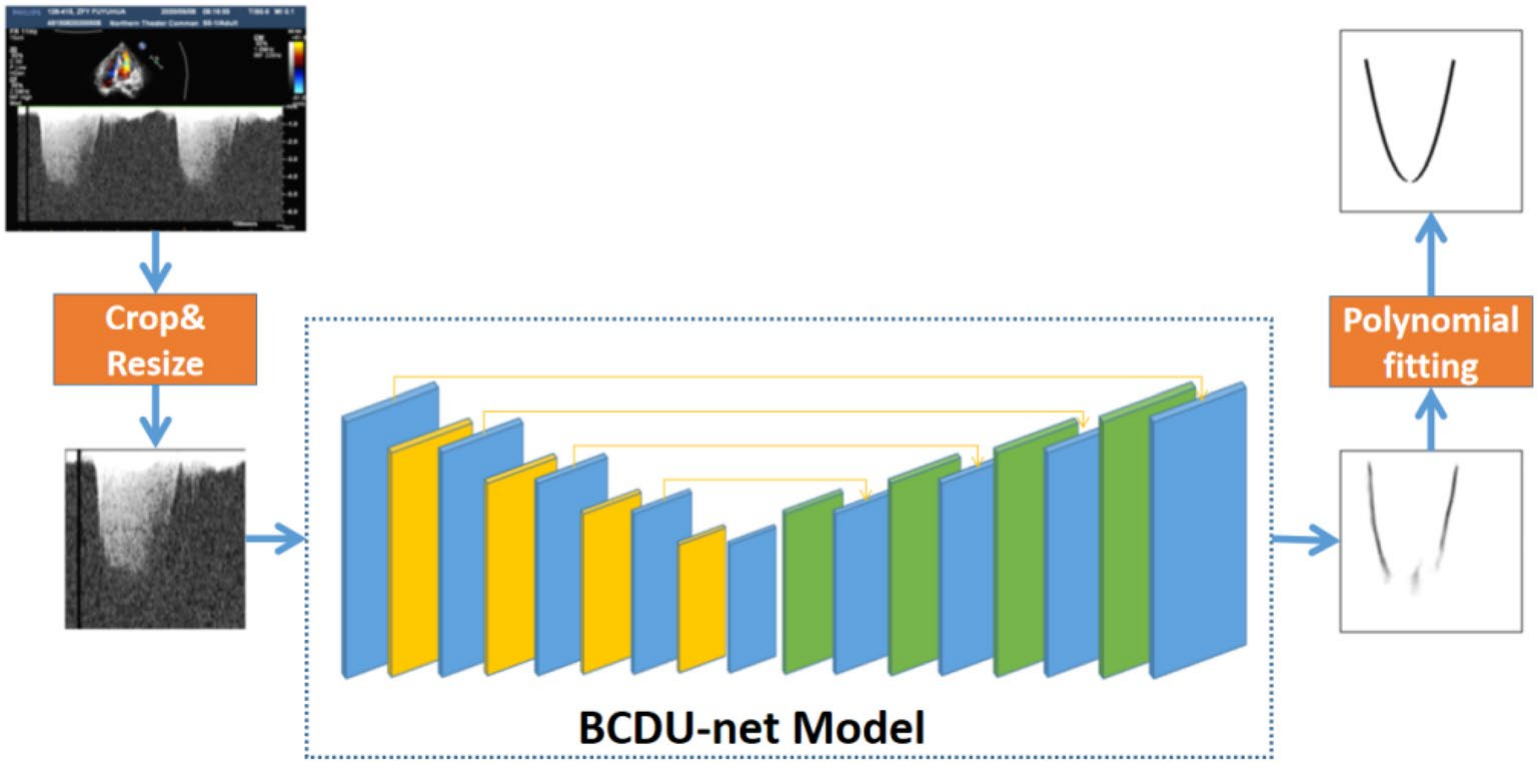
The overall structure of the deep learning network model

In order to accurately measure the intervals, *t*1–*t*2 and *t*1–*t*3, it is necessary to detect the spectrum edge firstly, so we proposed an intelligent method based on deep learning to complete this task. The method consists of two parts, a basic network for coarse detection and a post-processing module for refining. We adopted BCD-Unet deep learning model, which was proposed at the ICCV conference in 2019 [13], for edge detection firstly, but there are dislocation and fracture in the detection results. Therefore, we designed a post-processing module to deal with the above-mentioned problems. The post-processing module mainly uses the polynomial fitting method to refine the edges detected by BCD-Unet, making them clearer and smoother. The overall structure of the proposed method is shown as Fig. 2.

**Fig. 3:**
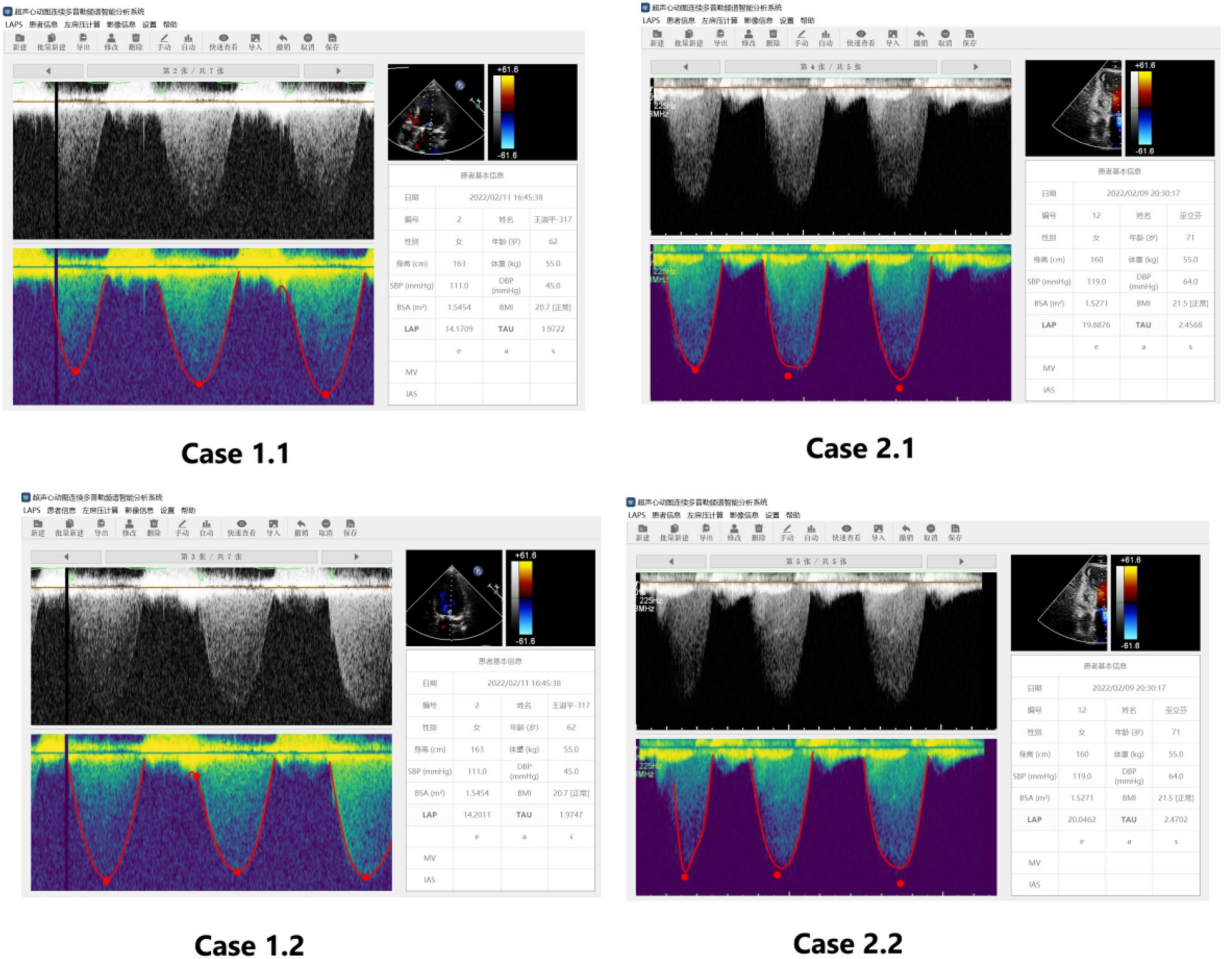
LAP_Ep_ measured by mitral regurgitation spectrum intelligent analysis system

We used the data collected by the hospital to train the model iteratively. The trained deep learning model can automatically detect the edge of mitral regurgitation spectrum. Then we encapsulated the model and built the system based on it. The system takes the mitral regurgitation spectrum as input and outputs the edge curve and LAP(directly a number), which is shown as Fig. 3. The software can only measure and calculate when the descending branch of the mitral regurgitation spectrum curve is complete, and the curve between at least 1 m/s and 3 m/s is good. When the peak value of the curve is less than 3 m/s, it will not be calculated.

### Statistical analysis

The statistical analysis and data visualization were conducted by SPSS 26.0 statistical software. Paired *t*-test was used to compare and analyze the measurement results of LAP_BP_ and LAP_Eq_ with LAP_C_ method. Meanwhile, correlation analysis was performed on the measurement results of LAP_BP_ and LAP_Eq_ with LAP_C_ method. The difference between LAP_BP_ and LAP_C_ was less than 10%, which was defined as the consistency between LAP_BP_ and LAP_C_, otherwise it was considered inconsistent. 28 patients were divided into two groups, 17 in the consistent group and 11 in the inconsistent group. The causes of inaccurate LAP_BP_ measurement were analyzed by single-factor analysis. The significant level is 0.05.

## Results

### Paired *t*-test of the measurement results of LAP_Eq_ and LAP_BP_ with LAP_C_ method

**Fig. 4:**
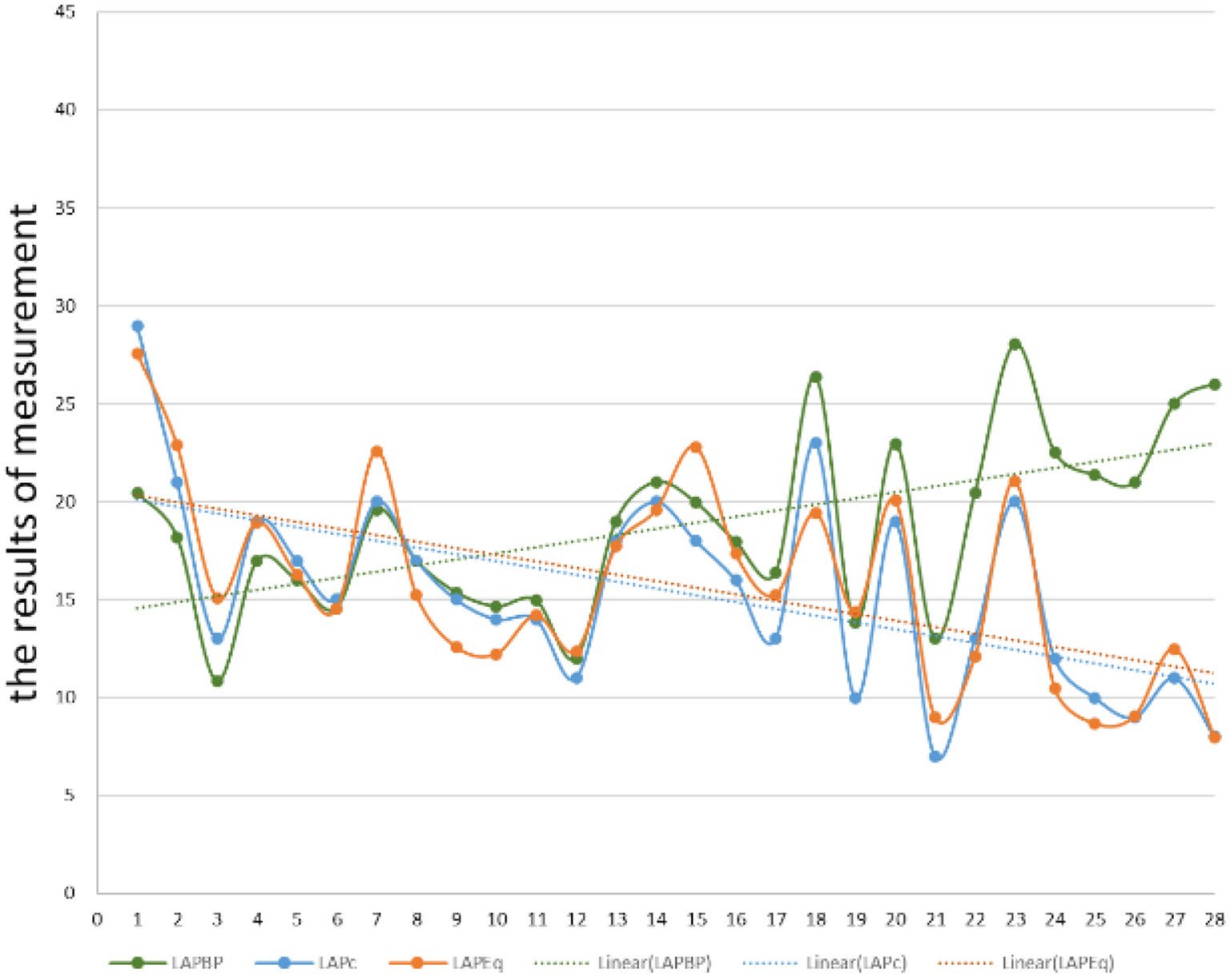
Paired *t*-test of the measurement results of LAP_Eq_ and LAP_BP_ with LAP_C_ method

A total of 95 mitral regurgitation spectra were obtained in 28 patients. LAP_C_, LAP_Eq_ and LAP_BP_ measured synchronously in 28 patients were visualized, as shown in Fig. 4. The average difference between LAP_C_ and LAP_Eq_ was 0.353, and the 95% confidence interval was (−1.112, 0.406). Paired *t*-test found no significant statistical difference between the measurement results of LAP_C_ and LAP_Eq_ (*t* = 0.954, *P* = 0.348). The average difference between LAP_C_ and LAP_BP_ was 3.332, and the 95% confidence interval was (−5.577, −1.087). Paired t-test found no significant statistical difference between the measurement results of LAP_C_ and LAP_BP_ (*t* = 3.045, *P* = 0.005). Although the measurement results of LAP_C_ and LAP_BP_ are consistent in some patients, there are significant differences between the two methods in the overall case as shown in Fig. 4.

### Correlation analysis of the measurement results of LAP_BP_ and LAP_Eq_ with LAP_C_ method

**Fig. 5:**
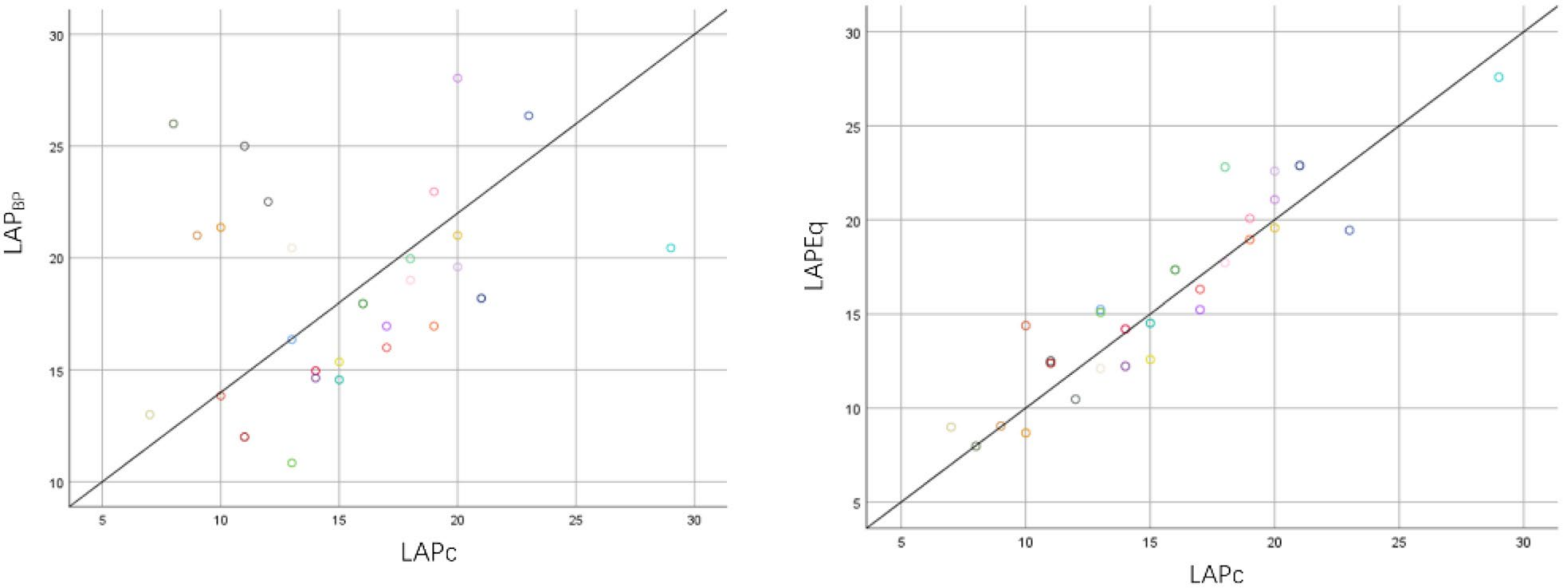
Correlation analysis of the measurement results of LAP_BP_ and LAP_Eq_ with LAP_C_ method

The correlation analysis of LAP_C_ and LAP_Eq_ shows that the results measured by the two methods are highly correlated and have significant statistical significance [*r* = 0.908(0.844,0.964), *P* < 0.001]. The correlation analysis of LAP_C_ and LAP_BP_ shows that the results measured by the two methods show a weak correlation, but they do not have significant statistical significance[*r* = 0.210, (−0.101, 0.510), *P* = 0.090] as shown in Fig. 5.

### Analysis of the difference between LAP_BP_ and LAP_C_ measurement

Whether there was atrial fibrillation or not had no significant effect on the measurement results of the two methods, *P* > 0.05. The analysis of disease composition found that the consistency rate of the two methods in patients with coronary heart disease and rheumatic heart disease without aortic stenosis was significantly higher than that in patients with mitral valve prolapse, *P* = 0.002 as shown in Table 1. In 11 MVP patients with P1 or P3 prolapse, the MR peak differential pressure was underestimated due to the MR severe eccentric direction. Seven MVP patients with A2 or P2 prolapse could accurately obtain the MR peak pressure difference and quantitatively measure LAP_BP_.

**Table 1:** Analysis of inaccurate LAP_BP_ measurement

Testvar	Consistent (*n* = 17)	Inconsistent (*n* = 11)	Statistic	*P*
Disease composition				
CABG and RHD	10(58.82)	0(0)	7.381	0.002
MVP	7(41.18)	11(100)		
Radial artery systolic pressure. mmHg	111(101,119)	102(96.5,107.5)	1.248	0.212
MR peak velocity. m/s	4.9(4.6, 5)	4.5(4.25, 4.72)	1.677	0.094
Sinus rhythm	11(64.71%)	5(45.45%)	0.452	0.441
Atrial fibrillation	6(35.29%)	6(54.55%)		

### Inter- and intra-observer variability of analysis

Intra- and inter-observer variability did not differ significantly in measuring LAP_BP_, *P*> 0.05. LAP_Eq_ measured with a special intelligent Doppler spectrum analysis software, and the intra- and inter-observer variability did not differ significantly, *P*> 0.05. Intra- and inter-observer agreement was well above 0.90 (*P*< 0.001) for all measures.

## Discussion

Accurate and real-time LAP measurement is an important factor in formulating and adjusting clinical treatment plans, but it is almost impossible to obtain LAP directly in clinical practice. Therefore, many critically ill patients can use PAWP instead of LAP_C_ through floating catheter. However, the application of floating catheter is also invasive and cannot be used routinely. A non-invasive method has been explored to evaluate left atrial pressure. The combined application of tissue Doppler of atrioventricular valve annulus, pre-mitral flow spectrum and pulmonary vein spectrum can be used for qualitative or semi-quantitative assessment of LAP [14, 15], and some can be used for quantitative assessment of LAP [16], but the correlation with LAP_C_ is only moderate [6, 17]. This study focused on the methodology of quantitative and real-time measurement of left atrial pressure using mitral regurgitation spectrum. We synchronously compared two methods of quantitative measurement of LAP using mitral regurgitation spectrum with PAWP, and analyzed the advantages and disadvantages of these two methods.

One is the early “Doppler + sphygmomanometer” method, that is, LAP_BP_ method, which uses the peak pressure difference of mitral regurgitation spectrum to measure the left atrial pressure quantitatively. The other has been developed by our team according to Weiss exponential equation and simplified Bernoulli equation. LAP_Eq_ is calculated by measuring any two intervals of the descending branch of the mitral regurgitation spectrum and bringing them into the equations. We collected the mitral regurgitation spectrum (TTE) and PAWP synchronously after placing the floating catheter before cardiac surgery, so that we can simultaneously use three methods to measure LAP. We compared the measurement results of LAPC with those of the other two methods, and analyzed the advantages and disadvantages of these methods and their clinical values.

It was found that LAP_BP_ method and LAP_C_ method have good correlation in some patients, such as ischemic heart disease, myocarditis, and patients without aortic stenosis [5]. By analyzing the data of patients with mitral valve prolapse, we found that LAP_BP_ and LAP_C_ were well correlated in 7 patients with A2 and/or P2 prolapse, while LAP_BP_ in 11 patients with P1 or P3 prolapse was lower than LAP_C_. Considering that the accuracy of LAP_BP_ is related to the mitral regurgitation angle and the regurgitation angle is related to the prolapse site, LAP_BP_ method is only applicable to some patients with mitral valve prolapse. To sum up, LAP_BP_ is a more practical method for quantitative measurement of LAP, which has better clinical application value in patients with central mitral regurgitation.

In theory, LAP_Eq_ method can measure LAP quantitatively in real time. However, the accuracy of LAP measurement depends on the accuracy of *t*1−*t*2 and *t*1−*t*3 measurement. In this study, the deep learning model of big data training was used to establish a software with automatic tracking envelope and automatic calculation capabilities to improve the repeatability of LAP_Eq_ measurement. The LAP_C_ of subjects in this study was between 8 and 29 mmHg. Paired *t*-test showed that there was no significant statistical difference between LAP_Eq_ and LAP_C_ (*t* = 0.954, *P* = 0.348), and there was a high correlation between the results of the two measurement methods (*r* = 0.908, *P* < 0.001), that is, LAP_Eq_ method can be used to measure LAP quantitatively in real time. The previous animal experiments [12] and our clinical trials have proved that the left atrial pressure can be measured quantitatively using mathematical equations, and our theoretical derivation is reasonable.

LAP_Eq_ method is less affected by other factors such as valve disease, systemic blood pressure, angle of mitral regurgitation, etc. The LAP_BP_ method is easily affected by the eccentric angle of the mitral regurgitation beam, and underestimates the peak pressure difference of MR. LAP_Eq_ method is used to measure the rate of left ventricular pressure decline. Whether the mitral regurgitation spectrum is eccentric has little influence on the accuracy of this method. However, it was found that LAP_Eq_ method needs to measure mitral regurgitation when the spectral edge of mitral regurgitation is clear enough, so it requires more mitral regurgitation than LAP_BP_ method. Therefore, we have continuously improved our measurement software. For patients with large mitral regurgitation, the software has good repeatability; for cases with relatively few mitral regurgitation, we first drew the edge manually, and then used the software to draw the mitral regurgitation spectrum curve to measure and calculate LAP, so as to improve the repeatability of this measurement method.

In addition, the LAP_Eq_ method derived from the mathematical formula is a direct quantitative measurement of LAP. In some cases, PAWP measured by floating catheter method is not equal to left atrial pressure, such as mechanical ventilation under PEEP, endotoxic shock [18], pulmonary embolism [19], ARDS, etc. Therefore, the application of PAWP instead of LAP in clinical practice is also limited. LAP_Eq_ method based on the descending branch of mitral regurgitation spectrum is a direct quantitative measurement of left atrial pressure, which has good repeatability and is worth popularizing.

To sum up, combining the advantages and disadvantages of various non-invasive methods for measuring left atrial pressure, we propose the following process for non-invasive quantitative measurement of left atrial pressure: first, use qualitative evaluation methods to determine the possibility of elevated left atrial pressure, and then decide which method to use for LAP measurement according to the amount of mitral regurgitation and the angle of regurgitation beam. LAP_Eq_ method can be used for patients with large regurgitation or eccentric regurgitation. For patients with small reflux, LAP_BP_ method can be considered for measurement. Repeated measurements are required to increase the repeatability of measurement and obtain more reliable left atrial pressure.

## Limitations

The application software used in LAP_Eq_ measurement in this study can only identify the Doppler spectrum of transthoracic echocardiography, but not that of transesophageal echocardiography. In addition, the sample number is small and does not include patients with moderate or severe aortic stenosis.

## Conclusions

This study shows that there is a good correlation between LAP_Eq_ and LAP_C_, which verifies that the non-invasive and direct quantitative measurement of left atrial pressure based on mitral regurgitation spectrum is feasible and has a good application prospect.

## Data Availability

Not applicable.
